# Relationship of Deoxynivalenol Content in Grain, Chaff, and Straw with *Fusarium* Head Blight Severity in Wheat Varieties with Various Levels of Resistance

**DOI:** 10.3390/toxins7030728

**Published:** 2015-03-05

**Authors:** Fang Ji, Jirong Wu, Hongyan Zhao, Jianhong Xu, Jianrong Shi

**Affiliations:** 1 State Key Laboratory Breeding Base of Food Quality and Safety in Jiangsu Province, Ministry of Agriculture, Nanjing 210014, China; E-Mails: jifang625@126.com (F.J.); yangzhouwjr@126.com (J.W.); hongyanz8910@126.com (H.Z.); xjh@jaas.ac.cn (J.X.); 2 Key Laboratory of Control Technology and Standardfor Agro-product Safety and Quality, Ministry of Agriculture, Nanjing 210014, China; 3 Key Laboratory of Agro-product Safety Risk Evaluation (Nanjing), Ministry of Agriculture, Nanjing 210014, China; 4 Institute of Food Quality and Safety, Jiangsu Academy of Agricultural Sciences, Nanjing 210014, China

**Keywords:** deoxynivalenol, FHB severity, wheat varieties

## Abstract

A total of 122 wheat varieties obtained from the Nordic Genetic Resource Center were infected artificially with an aggressive *Fusariumasiaticum* strain in a field experiment. We calculated the severity of Fusarium head blight (FHB) and determined the deoxynivalenol (DON) content of wheat grain, straw and glumes. We found DON contamination levels to be highest in the glumes, intermediate in the straw, and lowest in the grain in most samples. The DON contamination levels did not increase consistently with increased FHB incidence. The DON levels in the wheat varieties with high FHB resistance were not necessarily low, and those in the wheat varieties with high FHB sensitivity were not necessarily high. We selected 50 wheat genotypes with reduced DON content for future research. This study will be helpful in breeding new wheat varieties with low levels of DON accumulation.

## 1. Introduction

Fusarium head blight (FHB) is a fungal disease that affects numerous small grain species [1,2,3,4]. This disease is caused by members of the *Fusarium graminearum* species complex, which consists of at least 16 phylogenetically distinct species [5]. FHB reduces kernel set and kernel weight, which contribute to significant grain yield losses. The disease also decreases the nutritive and baking quality of grain through degradation of proteins. Mycotoxins may accumulate to unacceptable levels, making harvested grain and their byproducts unsuitable for human and animal consumption.

Deoxynivalenol (DON) is one of the most economically important trichothecenes produced by *F. graminearum*. It is stable at high temperatures and is soluble in water and some polar solvents [6]. DON is also referred to as vomitoxin, due to its ability to induce vomiting, especially in pigs [7]. In plants, DON delays seed germination and subsequent plant growth and development [8]. At the cellular level, the main toxic effect of DON is inhibition of protein synthesis via binding to ribosomes [9]. Additionally, DON acts as a virulence factor and plays an essential role in the spread of *F. graminearum* after the initial injection of a wheat plant [10].

Since the 1990s, interest in the health benefits and safety of cereal products has increased. Alimentary mycotoxin accumulation is a risk associated with the consumption of infected grain [11], and this has resulted in the establishment of maximum permissible DON levels for grain destined for human consumption. In China, the maximum permissible level of DON in cereals and their byproducts is 1 mg/kg [12].

Wheat straw and chaff are often used for feedstuff, but may be unsuitable for animal consumption if contaminated by Fusarium toxins. Kang and Buchenauer (2000) and Matthäus *et al.* (2004) reported that natural contamination of feed by Fusarium toxins resulted in altered nutrient composition and marked modification of cell wall compounds in the infected grain due to higher activities of protease, amylase, and several NSP-degrading enzymes [13,14]. K. Seeling *et al.* demonstrated that feeding animals with wheat and wheat chaff contaminated with the *Fusarium* toxins DON and zearalenone (ZON) caused slight changes in ruminal nutrient degradability and in rumen physiological parameters, probably due to fungus-related modifications of the grain [15].

The development of FHB-resistant cultivars has been effective in reducing damage caused by the disease, However, resistance to FHB is affected by the relationship between symptom intensity and toxin production [16,17,18]. Understanding the relationship between FHB and DON accumulation could be important in predicting the risks of mycotoxins in food, and may aid in the development of a more reliable and rational strategy for managing the FHB disease complex and reducing DON production. Numerous attempts have been made to relate disease incidence or severity to mycotoxin concentrations and the results have varied [19]. Despite the fact that most studies reported positive correlations between disease incidence and mycotoxin accumulation, the quantity of mycotoxins per unit of disease index differed considerably and some studies failed to find a significant relationship [20]. The warm and humid climate in Jiangsu Province, China, during wheat flowering is favorable for the development of FHB epidemics. In this study, 250 winter wheat varieties obtained from the Nordic Genetic Resource Center in Alnarp, Sweden, for FHB resistance in China (data not shown), we selected 122 varieties that exhibited stable type II resistance to examine the relationship between DON content and FHB severity and to screen for wheat genotypes with reduced DON content. In addition, we measured the concentrations of DON in the grain, chaff and straw of these varieties. Our results mayfacilitate the selection of varieties used for animal feed.

## 2. Results

### 2.1. FHB Severity in Wheat Cultivars

During an FHB epidemic in Jiangsu Province, China, in 2012 [21], the rate of infected spikelets was as low as 24% in the Sumai-3 cultivar, which exhibited the highest resistance, but was as high as 89% in susceptible cultivars. In this study, we found a large range of genetic variation among the varieties in the severity of FHB infection. The occurrence of infected spikelets was less than 25% in eleven varieties and greater than 89% in 26 varieties, and the remaining varieties exhibited intermediate levels of resistance to FHB (Table 1).

**Table 1:** *Fusarium* head blight (FHB) severity for selected wheat varieties from Sweden based on field-grown plants in 2012.

NO.	Accession name	Origin	PIS ^1^
1	Loyal	Sweden	31.69 ± 10.56
2	Hereford	Sweden	24.34 ± 7.64
3	Skagen	Sweden	23.35 ± 6.04
4	PAJ706-575A	Sweden	45.82 ± 22.35
5	Mariboss	Sweden	29.96 ± 22.27
6	Harnesk-7	Sweden	65.15 ± 11.67
7	IDUNA	Sweden	39.17 ± 16.00
8	ANKAR	Sweden	38.53 ± 20.22
9	ÅRING III	Sweden	100.00
10	EROICA	Sweden	22.61 ± 19.92
11	AROS	Sweden	33.08 ± 22.40
12	BANCO	Sweden	16.86 ± 8.73
13	NORRE	Sweden	12.19 ± 8.80
14	STURE	Sweden	56.28 ± 31.01
15	HELGE	Sweden	14.40 ± 0.73
16	LINNA	Sweden	29.39 ± 12.89
17	JYVÄ	Sweden	95.14 ± 6.82
18	SIGYN II	Sweden	100 ± 0.00
19	FOLKE	Sweden	32.61 ± 13.48
20	HOLGER	Sweden	24.12 ± 9.90
21	ALEMAR UST. HV. SG.	Sweden	61.48 ± 13.55
22	GUSALEK K.17	Sweden	96.07 ± 6.79
23	LANTVETE FRÅN UPPSALA	Sweden	100 ± 0.00
24	KOTTE	Sweden	53.70 ± 11.88
25	EXTRA SQUAREHEAD	Sweden	47.02 ± 11.75
26	RENODLAT SAMMETSVETE	Sweden	91.61 ± 6.20
27	THULE II	Sweden	92.81 ± 11.84
28	PANSAR II	Sweden	29.68 ± 6.63
29	SVEA I	Sweden	97.27 ± 6.10
30	PANSAR III	Sweden	79.84 ± 10.06
31	GYLLEN II	Sweden	85.29 ± 10.23
32	GLUTEN	Sweden	93.75 ± 12.50
33	BORG	Sweden	89.91 ± 14.39
34	PÄRL II	Sweden	37.50 ± 17.68
35	ROBUR	Sweden	26.34 ± 10.49
36	SEBA	Sweden	68.30 ± 11.44
37	VIRGO	Sweden	88.38 ± 18.02
38	SOLID	Sweden	40.82 ± 15.72
39	HILDUR	Sweden	17.09 ± 6.66
40	HANKKIJAN ILVES	Sweden	39.52 ± 10.72
41	Harnesk-5	Sweden	24.68 ± 9.89
42	GUSALEK K.10 A	Sweden	38.41 ± 23.08
43	KOSACK	Sweden	46.04 ± 11.00
44	MK 2-114	Sweden	74.57 ± 11.86
45	MK 2-302	Sweden	32.31 ± 6.84
46	MK 2-304	Sweden	85.23 ± 17.94
47	MK 2-306	Sweden	95.56 ± 9.94
48	MK 2-313	Sweden	27.27 ± 6.43
49	MK 2-502	Sweden	41.36 ± 10.15
50	MK 2-503	Sweden	100.00 ± 0.00
51	MK 2-506	Sweden	46.11 ± 16.35
52	MK 2-508	Sweden	31.11 ± 12.57
53	MK 2-542	Sweden	59.82 ± 15.41
54	MK 2-543	Sweden	83.43 ± 9.19
55	MK 2-547	Sweden	97.37 ± 5.26
56	MK 2-548	Sweden	21.67 ± 21.62
57	MK 2-556	Sweden	90.73 ± 11.73
58	MK 2-557	Sweden	77.06 ± 11.22
59	MK 2-558	Sweden	84.31 ± 13.25
60	MK 2-564	Sweden	63.92 ± 25.45
61	SALUT	Sweden	53.91 ± 12.70
62	ØSTBY	Sweden	30.81 ± 9.26
63	MK 2-501	Sweden	59.04 ± 11.22
64	MK 2-510	Sweden	94.18 ± 8.85
65	PORTAL	Sweden	30.01 ± 10.00
66	TJELVAR	Sweden	45.51 ± 9.95
67	TRYGGVE	Sweden	88.81 ± 10.62
68	MK 2-549	Sweden	79.50 ± 11.95
69	MK 2-566	Sweden	91.67 ± 14.43
70	MK 2-651	Sweden	42.84 ± 11.66
71	MK 2-656	Sweden	66.51 ± 28.36
72	MK 2-659	Sweden	39.18 ± 13.15
73	MK 2-660	Sweden	61.33 ± 21.33
74	MK 2-775	Sweden	44.96 ± 7.58
75	MK 2-780	Sweden	88.18 ± 21.70
76	MK 2-786	Sweden	86.93 ± 15.12
77	MK 2-787	Sweden	44.74 ± 12.32
78	PANU	Sweden	65.90 ± 34.88
79	KONGE III	Sweden	37.50 ± 15.52
80	TYSTOFTE SMAAHVEDE	Sweden	34.03 ± 10.88
81	RENTAL	Sweden	57.33 ± 13.20
82	STAVA	Sweden	53.88 ± 15.44
83	RUDOLF RUBIN	Sweden	90.58 ± 13.79
84	KIRSTEN	Sweden	42.80 ± 11.91
85	LONE	Sweden	43.68 ± 8.71
86	BRANDT	Sweden	40.56 ± 11.73
87	KARAT	Sweden	39.61 ± 17.13
88	MK 2-101	Sweden	41.43 ± 17.05
89	MK 2-113	Sweden	49.57 ± 24.98
90	MK 2-116	Sweden	71.81 ± 24.64
91	MK 2-122	Sweden	94.44 ± 13.61
92	MK 2-130	Sweden	29.91 ± 10.39
93	MK 2-316	Sweden	47.09 ± 15.38
94	MK 2-317	Sweden	69.01 ± 13.76
95	MK 2-337	Sweden	64.83 ± 15.38
96	MK 2-567	Sweden	57.99 ± 39.13
97	MK 2-655	Sweden	68.97 ± 26.56
98	MK 2-138	Sweden	32.40 ± 16.47
99	MK 2-679	Sweden	81.00 ± 7.12
100	MK 2-788	Sweden	70.70 ± 10.31
101	MK 2-847	Sweden	25.99 ± 13.17
102	Saxild	Sweden	37.17 ± 18.92
103	Abba	Sweden	33.86 ± 4.74
104	Konsul	Sweden	100.00 ± 0.00
105	Rektor	Sweden	35.89 ± 8.00
106	Stakado	Sweden	100.00 ± 0.00
107	Kerimäkeläinen	Sweden	95.45 ± 9.09
108	Sampo	Sweden	95.83 ± 9.32
109	Väinö	Sweden	87.20 ± 11.37
110	Speltti Vaalea	Sweden	67.79 ± 19.61
111	Speltti Ruskea Baulander	Sweden	56.30 ± 18.53
112	Kökar	Sweden	31.96 ± 8.09
113	Olympia	Sweden	77.45 ± 13.20
114	H 8703	Sweden	100.00 ± 0.00
115	H 8196	Sweden	100.00 ± 0.00
116	H 8202	Sweden	100.00 ± 0.00
117	H 8222	Sweden	76.78 ± 35.98
118	H 8296	Sweden	34.72 ± 28.69
119	H 8300	Sweden	88.75 ± 15.56
120	H 8305	Sweden	19.57 ± 7.03
121	H 8310	Sweden	94.44 ± 9.62
122	H 8311	Sweden	33.55 ± 7.08
-	Control cultivar	-	-
-	Annong 8455	China	89.65 ± 2.33
-	Sumai 3	China	24.21 ± 9.04

^1^ PIS is the percentage of infected spikelets.

### 2.2. Deoxynivalenol (DON) Levels in Wheat Grain, Straw, and Glume Tissues after Artificial Inoculation of Wheat Ears

**Figure 1:**
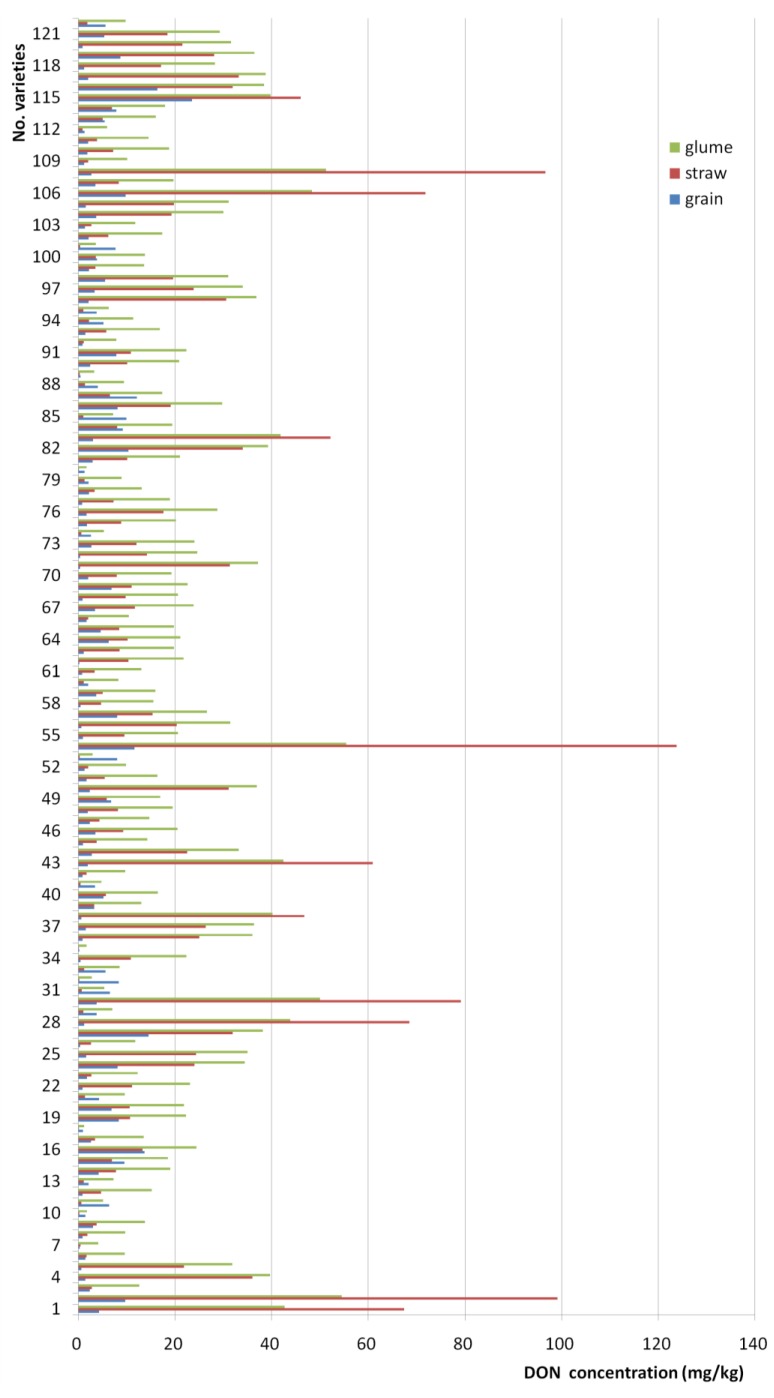
Distribution of deoxynivalenol in grain, glume and straw of wheat with different varieties.

A total of 122 wheat varieties were inoculated, and DON levels were determined in a total of 366 grain, straw, and glume samples. DON content was higher in glumes than in straw in 90.16% of the samples, and higher in straw than in grain samples in 73.77%. The DON content was higher in glumes than in grain in 95.90% of the samples (Figure 1). One-way analysis of variance (ANOVA) showed significant differences in DON content among grain, straw, and glumes (*F* = 42.20, *p* < 0.0001). The different plant sample types showed positive correlations (*r*_grain *vs.* straw_ = 0.261 **, *r*_grain *vs.* glume_ = 0.251 **, and *r*_straw *vs.* glume_ = 0.879 **). Although differences in mycotoxin content were large, the distribution of DON in plant tissues exhibited a distinct pattern (Table 2).

**Table 2:** Deoxynivalenol (DON) levels in samples of various wheat tissues determined using High Performance Liquid Chromatography-tandem Mass Spectrometry.

DON (mg/kg)					
Tissue	*n*	Mean *	Median	Max	Min
grain	122	3.88 ^A^	2.38	23.46	0.16
straw	122	15.30 ^B^	7.85	123.74	0.04
glume	122	20.95 ^C^	19.13	55.42	1.15

*: Values in the same column with different capital letters were significantly different (α = 0.01); Different letters within a column represent significant difference at *p* = 0.01.

### 2.3. DON Levels in Tissues from Wheat Varieties Exhibiting Differing FHB Disease Severity

**Figure 2:**
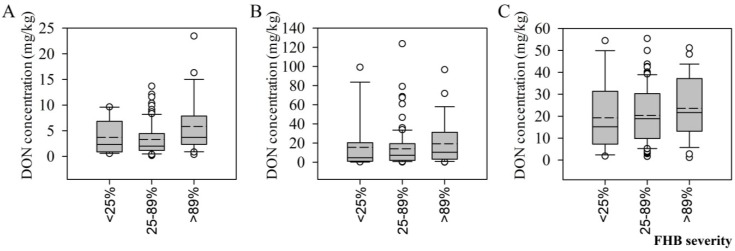
Box plots of the distribution of deoxynivalenol (DON) levels in wheat tissues ((**A**): grain; (**B**): straw; (**C**): glumes) inoculated with *Fusarium graminearum* at various levels of Fusarium head blight disease severity. Solid and dashed lines indicate medians and means, respectively. The box boundaries indicate the 75% and 25% quartiles. The whisker caps indicate 90th and 10th percentiles and the circles indicate the 95th and 5th percentiles.

To better understand how the level of DON contamination varied with FHB severity, we divided the entire dataset into three levels of FHB severity relative to the severity of FHB according to the control cultivars (Figure 2): <25% (*n* = 11, mean = 20.08%), 25%–89% (*n* = 85, mean = 52.94%), and >89% (*n* = 26, mean = 96.30%).

The DON contamination levels did not increase consistently with increasing FHB severity (Figure 2). The average and median DON levels were the highest in all three wheat tissues when the FHB severity was greater than 89%. In the grain and the straw, the average and median DON levels were lowest when the FHB severity was between 25% and 89%, whereas the lowest average and median levels in the glume occurred when the FHB severity was below 25%. In general, the DON levels in the wheat varieties that were highly resistant to FHB were not necessarily low, and the DON levels in the varieties that were highly sensitive to FHB were not necessarily high.

### 2.4. Relationship between FHB Severity and DON Levels among Wheat Varieties and Selection of Wheat Genotypes with Reduced DON Content

To further compare the variation of DON contamination levels with FHB severity among wheat varieties, the data were allocated into four groups according to average FHB severity and DON contamination level: (1) varieties with low FHB severity and low DON contamination; (2) varieties with low FHB severity and high DON contamination; (3) varieties with high FHB severity and low DON contamination; and (4) varieties with high FHB severity and high DON contamination (Figure 2). The horizontal and vertical lines represent the *y*- and *x*-axis average values, respectively.

**Figure 3:**
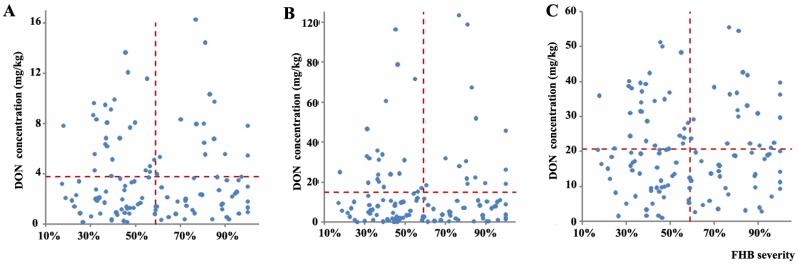
Relationship between Fusarium head blight (FHB) severity and deoxynivalenol levels in wheat tissues ((**A**): grain; (**B**): straw; (**C**): glumes). Dashed lines indicate the average FHB severity (59%) and average concentration of DON ((**A**): 3.78 mg/kg, (**B**): 14.77 mg/kg, (**C**): 20.65 mg/kg) in 366 samples (grain samples = 122, straw samples = 122, glume samples = 122).

The relationship between DON content in grain and FHB severity is shown in Figure 3A. Groups 1, 2, 3, and 4 included 45, 25, 36, and 16 varieties, respectively. For the straw samples of the 122 wheat varieties, 41% were in group 1, 16% in group 2, 30% in group 3, and 13% in group 4 (Figure 3B). For the glume samples, 33% were in group 1, 25% in group 2, 26% in group 3, and 16% in group 4 (Figure 3C). Many of the samples exhibited low DON contamination levels (groups 1 and 3) and may correspond to varieties that would be useful for breeding new wheat varieties with low levels of DON accumulation.

Among the 122 varieties, 27 were consistently in group 1, six were consistently in group 2, 23 were consistently in group 3, and seven were consistently in group 4. For both straw and glumes, 13 varieties were consistently in group 1, 14 were consistently in group 2, nine were consistently in group 3, and nine were consistently in group 4. For both straw and grain, four varieties were consistently in group 1 and four varieties were consistently in group 3. For both grain and glumes, only six varieties were consistently in group 2.The varieties in group 1 had low DON contamination levels not only in the grain but also in the straw and glume. These varieties were Skagen, IDUNA, ANKA, EROICA, BANCO, NORRE, ROBUR, HILDUR, Harnesk-5, MK 2-302, MK2-313, MK 2-506, MK 2-508, MK 2-547, SALUT, MK 2-549, MK 2-775, MK 2-787, KONGE III, TYSTOFTE SMAAHVEDE, MK 2-316, MK 2-317, MK 2-567, Abba, Speltti Vaalea, Kökar, and Olympia.

The varieties in group 3 had low DON contamination levels in all three wheat tissues while exhibiting high FHB severity. These varieties were Harnesk-7, ÅRING III, JYVÄ, SIGYN II, LANTVETE FRÅN UPPSALA, RENODLAT SAMMETSVETE, SVEA I, GUSALEK K.10 A, MK 2-304, MK 2-306, MK 2-557, MK 2-558, MK 2-564, MK 2-501, TJELVAR, MK 2-651, MK 2-780, PANU, MK 2-113, MK 2-788, Konsul, Sampo and Speltti Ruskea Baulander. We selected these 50 varieties as wheat genotypes with reduced DON content for future studies.

## 3. Discussion

Resistance to FHB can be characterized as consisting of two components: resistance to initial penetration and resistance to pathogen spread in the host tissue [22]. In addition to the resistance mechanisms that determine the severity of FHB, other mechanisms may influence DON content in kernels, including degradation or conjugation of DON and tolerance to DON [8,23,24]. DON tolerance results in an increased level of resistance and inherent prevention of trichothecene accumulation [25]. DON production is an important factor in the overall pathogenesis of FHB. Several studies have reported a significant positive correlation between the incidence of FHB and DON concentration [20,26]. Snijders and Perkowski reported that reduced disease incidence in the field ensured reduced mycotoxin content of the grain [8]. Correlations between FHB ratings and DON content were high in segregating material and in a collection of varieties with different levels of FHB resistance [27,28]. Others have demonstrated a significant correlation between DON and the fungal biomass of the grain [18,20,29]. These results suggest that new cultivars could be selected based on disease symptoms to ensure low levels of DON.

Bai *et al.* identified some wheat cultivars with severe FHB symptoms and low DON levels, particularly in cultivars with moderate resistance to *F. graminearum* [20]. Chen *et al.* inoculated five wheat varieties with a strain of *F. graminearum* and found no correlation between DON concentration and FHB severity [30]. Liu also found no correlation between DON concentration and FHB severity in wheat, barley (*Hordeum vulgare* L.) and oats (*Avena sativa* L.) after inoculation with a complex of *Fusarium* species [23]. In natural contamination, there was also no correlation between FHB severity in wheat and DON concentration or between the presence of *Fusarium* and DON concentration [31,32,33]. These differences in results may be due to the differences in the genotypes planted, pathogen populations, weather conditions, or management practices among the various studies.

Certain genotypes can limit the development of mycelium in the grain, thereby protecting it from degradation and limiting the visual signs of attack, but those genotypes often have low tolerance to mycotoxins [20]. Conversely, other cultivars may exhibit severe FHB symptoms and low mycotoxin levels. Beyer *et al.* studied the moderately susceptible wheat cultivar Dekan, which can partially avoid infection and then limit DON accumulation in the kernels when infections cannot be avoided [34].

We found DON contamination to be highest in glumes, intermediate in the straw, and lowest in the grain. DON produced by FHB in wheat can also have physiological effects in other parts of the plant [25]. Between the chaff and the kernel, several physical barriers could potentially limit the movement of the fungus and therefore prevent DON to move from the point of initial infection on the glumes or chaff to the kernel [35]. Snijiders *et al.* and Doohan *et al.* reported the ability of the toxin to translocate from chaff to the grain [36,37]. Data from previous studies suggest that the highest accumulation of mycotoxins in the barley hull [38]. Legzdina *et al.* reported that hulless barley tends to be less prone to mycotoxin contamination than covered barley, assuming a similar level of field FHB severity [39]. Wang and Miller reported higher levels of DON in the chaff than in the kernel of wheat [40].

In mature plants, DON appears to circulate in the phloem, with concentration following a descending gradient from the rachis, through the lemmas and grain, to the peduncle [41]. In our results, we found that the DON content was lowest in grain, then in straw and highest in glume. Schroeder and Christensen injected spores into the rachis and showed that the pathogen was able to migrate within the plant and propagate more rapidly longitudinally than transversely. Contaminated straw and glume residues remaining on the soil surface can infect seedlings of the following crop during the vegetative growth stage [42].

In conclusion, understanding the relationship between FHB severity and mycotoxin contamination is important. The conditions under which this relationship is purely qualitative rather than quantitative are unclear. In China, the increased use of high-yielding wheat varieties with greater susceptibility to FHB has increased the incidence of mycotoxins, indicating that the goal of a breeding program should be to select for both mycotoxin resistance and grain yield.

## 4. Materials and Methods

### 4.1. Reagents and Chemicals

DON was obtained from Sigma-Aldrich (Shanghai, China) at a concentration of 100 µg/mL in acetonitrile. HPLC-grade acetonitrile and methanol were obtained from Merck (Darmstadt, Germany). Deionized water (<8 MΩ cm^−1^ resistivity) was produced using a Milli-Q water purification system (Millipore, Bedford, MA, USA). All solvents were passed through 0.22-µm cellulose filters (Jinteng, Tianjin, China) before use.

### 4.2. Plant Material

Winter wheat seeds were obtained from the Nordic Genetic Resource Center in Alnarp, Sweden. Sumai-3 (high resistance) and Annong-8455 (high susceptibility) cultivars were used as control varieties to define levels of FHB resistance [43,44].

Winter wheat was grown according to current recommendations of integrated pest management, although applications of fungicide against *Fusarium* species were omitted. The field experiment was conducted at the Luhe experimental station, Jiangsu Academy of Agricultural Science (Luhe), Nanjing, China (32°28.583′N, 118°38′E) in 2011. Wheat was sown in late October in rows 150 cm in length and 33 cm apart with three replicates using a randomized block design.

An aggressive strain, F0613, was used to produce macroconidia in mung bean extraction liquid medium, as previously described [45]. The F0613 strain was isolated originally from diseased wheat grain in 2006 in Jiangsu Province. It belongs to *Fusarium asiaticum*, a member of the *Fusarium graminearum* species complex. At the heading stage, the middle spikelets of wheat flowers were inoculated with 10 μL of the conidial suspension containing 1 × 10^5^ spores mL^−1^. Twenty days after inoculation, the numbers of infected spikelets and spikelets per head were recorded. The percentage of infected spikelets was calculated for each head. The mean FHB severity of 50 heads was calculated and all the 50 heads were pool into one sample for further analysis [46].

### 4.3. Mycotoxin Analysis

#### 4.3.1. Preparation of Analytical Standard Solutions

Individual stock standard solutions were prepared as prescribed by the manufacturer. The working dilution for the calibration curve was prepared using a sample extract from a blank wheat sample (matrix-matched standard curve).

#### 4.3.2. Sample Preparation

Harvested grain samples were cleaned manually. The glume was separated from the kernel. Straw was chopped into 3.0-cm segments that were subsequently pulverized. The grain was ground to a powder of 20-meshe fineness in a laboratory mill (Ika Werke, Staufen, Germany), and the chaff and straw were treated with liquid nitrogen and pulverized using a Moulinette 320-grinder (Moulinex, Barcelona, Spain). All samples were stored at 4 °C for a maximum of 7 day.

The finely ground samples (10 g) were weighed and extracted with 40 mL of acetonitrile:water:acetic acid (79:20:1 *v*/*v*/*v*) at 180 rpm for 30 min [47].

After centrifugation at 3000 rpm for 10 min, 0.5 mL of each final extract was diluted with acetonitrile:water:acetic acid (20:79:1, *v*/*v*/*v*) and filtered through a nylon filter (13 mm in diameter, 0.22-µm pore size) into an autosampler vial, capped and analyzed by LC-MS/MS [48].

#### 4.3.3. LC-MS/MS Analysis

The LC-MS/MS system consisted of an Agilent 1200 HPLC, an Agilent 6410B triple-quadrupole mass spectrometer, and an Agilent MassHunter Workstation running Qualitative Analysis Version B.01.03 software (Agilent, Shanghai, China, 2001) for data acquisition and analysis. The analytical column was an XDB-C18 (2.1 × 150 mm, 3.5-µm bead diameter; Agilent, Shanghai, China) column and the column temperature was held constant at 30 °C. Nitrogen was used as a drying gas at 10 L/min. The capillary voltage was 4 kV, the nebulizer pressure was 25 psi, and the drying gas temperature was 350 °C. Mycotoxins were analyzed via multiple reaction monitoring (MRM). Mass spectrometric parameters of the mycotoxins and the composition of the mobile phase have been described by Soleimany *et al.* [49].

Quantification was performed against a matrix-matched calibration curve. During method development, limits of detection (LOD) and quantification (LOQ) scores for each analyte were determined on the basis of the signal-to-noise rations of 3:1 and 10:1. The LOD and LOQ scores were 10 and 20 µg/kg, respectively.

### 4.4. Statistical Analysis

All data are expressed as percentages or means ± relative standard deviation (RSD) using MS Excel.
